# A Pilot Study Evaluating the Effect of Artesunate on Glymphatic‐Related Fluid Transport in Parkinson's Disease Mouse Models In Vivo and Ex Vivo

**DOI:** 10.1002/cns.71145

**Published:** 2026-09-16

**Authors:** Wan‐ting Han, Wen‐hui Lu, Hui Xu, Guang‐hui Xie, Yan Cao, Xin‐ying Wu, Fan Qiu, Tong Fu

**Affiliations:** ^1^ Department of Radiology, Nanjing First Hospital Nanjing Medical University Nanjing China; ^2^ Department of Radiology, Shanghai Pudong Hospital Fudan University Affiliated Pudong Medical Center Shanghai China; ^3^ Department of Nuclear Medicine, Nanjing First Hospital Nanjing Medical University Nanjing China

**Keywords:** AQP4, artesunate, glymphatic system, Parkinson's disease, α‐Synuclein

## Abstract

**Aims:**

Artesunate (ART) has shown neuroprotective potential, but its effects on glymphatic‐related fluid transport in Parkinson's disease (PD) remain unclear. This pilot study aimed to explore whether ART affects glymphatic‐related fluid transport in a PD mouse model and to assess the feasibility of evaluating these changes in vivo.

**Methods:**

C57BL/6 WT and A53T mice (*n* = 6 each) were divided into four groups (WT, WT + ART, A53T, and A53T + ART) and treated with ART or DMSO for 7 days. Glymphatic‐related fluid transport was assessed by dynamic MRI after intraventricular Gd‐DTPA injection. Immunofluorescence evaluated α‐syn, AQP4, and AQP4‐GFAP colocalization, while transmission electron microscopy (TEM) assessed astrocytic end‐feet integrity.

**Results:**

ART was associated with regionally heterogeneous changes in tracer‐related MRI signal dynamics in both WT and A53T mice. In PD mice, ART was associated with partial restoration of AQP4 polarization in selected brain regions and improved perivascular astrocytic end‐feet morphology, accompanied by reduced α‐syn immunofluorescence signal in the substantia nigra.

**Conclusion:**

ART was associated with regionally heterogeneous changes in glymphatic‐related fluid transport dynamics and AQP4 polarization in this pilot PD mouse study. These preliminary findings support further investigation of ART as a potential modulator of brain fluid transport‐related pathways in PD.

## Graphical abstract

Artesunate (ART) treatment was associated with enhanced glymphatic CSF‐ISF exchange in both wild‐type (WT) and A53T mouse models of Parkinson's disease, with regional differences observed by dynamic MRI. In A53T mice, ART was associated with restoration of AQP4 polarization and reduced α‐syn levels in the SN. TEM confirmed that ART reversed astrocytic end‐feet detachment. These results suggested that ART has a potentially therapeutic role in modulating glymphatic function.

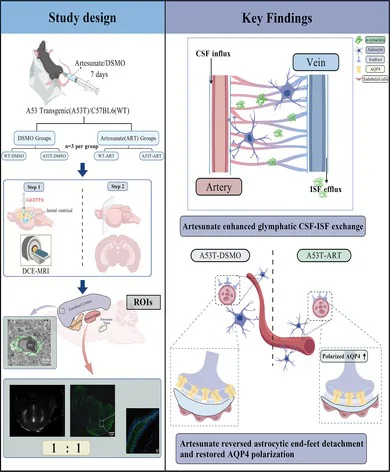

## 1 Introduction

Parkinson's disease (PD) is a progressive neurodegenerative disorder characterized by degeneration of dopaminergic neurons in the substantia nigra pars compacta (SNpc) and abnormal aggregation of α‐synuclein (α‐syn) [1]. The accumulation of α‐syn, particularly in the SNpc, is thought to trigger neuronal degeneration of dopaminergic neurons, suggesting that an imbalance between α‐syn aggregation and clearance plays a pivotal role in PD pathogenesis and progression [2].

The glymphatic system is a brain‐wide waste clearance pathway that facilitates the removal of interstitial solutes such as α‐syn by enabling cerebrospinal fluid (CSF) to enter the brain via periarterial spaces, mix with interstitial fluid (ISF), and promote solute clearance. The ISF, carrying metabolic waste, drains along perivenous spaces and exits through meningeal and cervical lymphatics into the venous circulation. Thus, reduced efficiency in CSF‐ISF exchange may impair α‐syn clearance, contributing to its pathological accumulation in PD [3, 4].

Aquaporin‐4 (AQP4), which is primarily located on astrocytic end‐feet surrounding cerebral vessels, is essential for glymphatic function by facilitating CSF‐ISF exchange. AQP4 promotes solute clearance, including α‐syn, via perivascular pathways. Disruption of AQP4 expression or polarization impairs this process [5, 6]. In PD models, AQP4 downregulation and mislocalization are linked to glymphatic dysfunction and α‐syn accumulation [7]. Moreover, reduced AQP4 mRNA levels in the blood of PD patients [8], along with a negative correlation between AQP4 and α‐syn in the temporal neocortex in postmortem examinations [9], suggest that AQP4 dysregulation contributes to impaired glymphatic clearance of α‐syn in PD and highlights AQP4 as a potential therapeutic target.

Artesunate (ART), a well‐characterized derivative of artemisinin with favorable water solubility and bioavailability [10], can readily cross the blood–brain barrier (BBB) [11]. ART exhibits low toxicity, potent anti‐inflammatory effects, and the ability to modulate the neuroinflammatory response properties, thereby extending its therapeutic relevance beyond parasitic infection [12, 13, 14, 15]. In PD mouse models, ART has been shown to exert a protective effect on dopaminergic neurons [16] and improve motor functions [17]. However, whether ART directly modulates the glymphatic system in PD remains unclear. Moreover, previous studies on ART's effects in PD models lack objectively observable in vivo evidence, limiting their clinical translational potential and applicability.

To investigate whether ART modulates glymphatic‐related fluid transport in a PD mouse model using in vivo dynamic MRI, we evaluated CSF‐ISF exchange‐related dynamics by tracking the distribution of the contrast agent Gd‐DTPA from the left lateral ventricle into the brain parenchyma using dynamic in vivo MRI, following established protocols in mouse models [18]. Ex vivo measures of α‐syn burden, AQP4 expression/polarization, and astrocytic end‐feet integrity were used for supportive correlation.

## 2 Materials and Methods

### 2.1 Animals and Drugs

All efforts were made to minimize animal suffering and the number of animals used [19]. Male wild‐type (WT) (*n* = 6) and A53T transgenic mice (*n* = 6) aged 14 weeks were obtained from Cavens (Changzhou Cavens Laboratory Animal Technology, China). The A53T group assignment was based on the established transgenic genotype provided by the supplier. No additional behavioral phenotyping was performed in the present study. All animals were housed in an air‐conditioned room under a 12‐h/12‐h light/dark cycle (lights on, 8:00 am through 8:00 pm). Food and water were provided ad libitum. WT and A53T mice were randomly allocated into two distinct cohorts each, yielding a total of four experimental groups: the WT, WT + ART, A53T, and A53T + ART groups. As this was a pilot exploratory study, group sizes were small and were intended primarily to assess feasibility and generate preliminary observations for future larger‐scale validation. The mice in the artesunate treatment groups were administered artesunate (Sigma, St. Louis, MO, USA) at a dose of 5 mg/kg, dissolved in DMSO, via intraperitoneal injection once daily at the same time each day for 7 consecutive days. DMSO was administered to both WT and A53T groups as the vehicle control. This 7‐day treatment duration was selected as a short‐term exploratory intervention to assess whether ART was associated with measurable changes in MRI‐based parameters and ex vivo histological/ultrastructural readouts within a defined experimental timeframe. The present study was not designed to compare multiple treatment durations; therefore, the 7‐day regimen should not be interpreted as an optimized treatment schedule.

### 2.2 Dynamic Contrast‐Enhanced MRI

**FIGURE 1:**
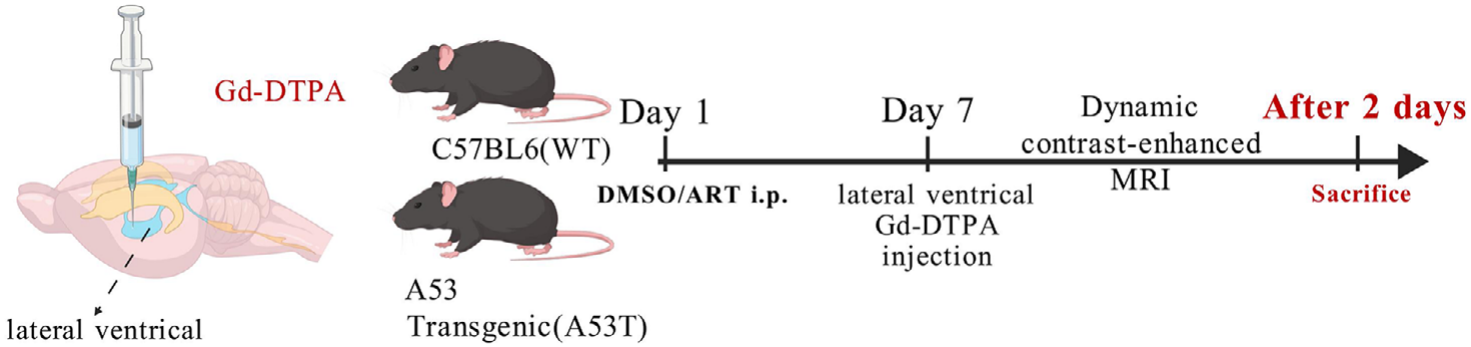
Experimental protocol of dynamic contrast‐enhanced MRI. Baseline scans were acquired before Gd‐DTPA injection. The figure was created with BioGDP.com.

To explore brain CSF‐ISF exchange‐related dynamics in vivo, intraventricular delivery of Gd‐DTPA and serial T1‐weighted MRI acquisition were performed. The protocol for MR scanning (Figure 1) was based on a previous report [20] and is described in detail as follows:

### 2.3 Surgical Preparation

Mice were anesthetized with 0.3% sodium pentobarbital and positioned in a stereotaxic frame. A vertical incision was made using ophthalmic scissors, starting approximately 1.5 cm above the ear, and extending toward the nose, continuing downward vertically to approximately 0.5 cm below the ear, and then vertically into the center of the mouse's head to expose the bregma. Based on the fifth edition of Paxinos and Franklin's “The Mouse Brain in Stereotaxic Coordinates” [21], the bregma and the interaural line were employed as reference points to establish the zero point and determine the injection coordinates. Once the bregma was exposed, a 50 μL glass microsyringe was used to inject 10 μL of a low‐molecular‐weight paramagnetic contrast agent (38 mM Gd‐DTPA, molecular weight 938 Da, in filtered 0.9% NaCl) at a rate of 1 μL/min into the left lateral ventricle (anterior‐posterior, −0.3 mm, medial‐lateral, 1.0 mm, dorsal‐ventral, 2.5 mm) [18].

### 2.4 MRI Acquisition

Anesthesia was maintained with a mixture of O2 and 2% isoflurane at a flow rate of 400 mL/min. During MR image acquisition, the isoflurane concentration was initially set at 2% and then adjusted as needed to maintain a stable respiratory rate of 100 ± 10 breaths per minute. Additionally, a thermoregulation system was employed to maintain body temperature at 37.0°C–37.5°C. Following the acquisition of MR images, the mice were individually placed on heated pads for recovery, marked for identification, and then returned to their respective cages.

All imaging was performed with a 7.0‐T VNMRS horizontal‐bore MRI scanner (Siemens, Germany). A T1‐weighted gradient‐echo sequence was used to detect the dynamic flow of the Gd‐DTPA with the following parameters: repetition time = 300 ms, echo time = 8.9 ms, flip angle = 90°, number of acquisitions = 1, matrix size = 256 × 256, and scanning time = 6 min. Baseline scans were acquired before Gd‐DTPA injection. Magnetic resonance images were continually acquired for a total of 60 min, with acquisitions conducted at 5 min, 15 min, 30 min, 45 min, and 60 min following the administration of Gd‐DTPA, respectively.

### 2.5 Image Processing and Analysis

The substantia nigra (SN), striatum, hippocampus, and caudal cortex were chosen as regions of interest (ROIs) for their relevance to PD pathology and glymphatic‐related fluid transport. The SN and striatum are central to motor symptoms and α‐syn accumulation [1, 22], while the hippocampus reflects cognitive impairment and AQP4 dynamics [23, 24]. The caudal cortex serves as a comparative region with distinct astrocytic features [25]. Together, these regions allow spatially resolved evaluation of ART's impact on glymphatic‐related fluid transport. The ROIs were drawn based on Paxinos and Franklin's mouse brain atlas [21]. The mean signal intensity within these ROIs was measured using the open‐source software ITK‐SNAP (version 4.0.2; available at http://www.itksnap.org). For analysis of the regional CSF‐ISF exchange‐related dynamics, the mean signal intensity ratio was calculated for each time point using the following expression:

$$\mathrm{MR}\;\text{Intensity Ratio}=I\left(\mathrm{ROI}\right)/I\left(\mathrm{BG}\right)$$

where *I* refers to the mean signal intensity, ROI refers to the selected regions of interest, and the *BG* (background) refers to the intensity of the subcutaneous muscle of the cranium located at the same slice as the corresponding ROI.

### 2.6 Immunofluorescence

Two days after the MR experiment was concluded, the mice were euthanized with an overdose of sodium pentobarbital (10 mL/kg, intraperitoneally), with fresh brain tissue harvested within 30 min. The brains were fixed in 4% paraformaldehyde. After permeabilization and blocking, the brain slices were incubated overnight at 4°C with the following primary antibodies: anti‐AQP4 (1:200, Ab259318, Abcam), anti‐GFAP (1:600, 3670 T, Cell Signaling Technology), and anti‐Syn (1:5000, Ab280377, Abcam). The next day, the slices were incubated with secondary antibodies for 1 h at room temperature.

Three brain slides per mouse were analyzed, with three fields of view per slide using a confocal microscope and a VS200 virtual slide microscope. Photomicrographs were analyzed using ImageJ software (ImageJ v1.53a, USA, http://rsb.info.nih.gov/ij/, 1997–2011). Three ROIs were bilaterally delineated on each brain image slice encompassing the target region (e.g., SN) from each mouse that underwent the MRI scans using ImageJ while blinded to animal group. Signal intensity measures for α‐syn and AQP4, (thresholded for immunoreactivity) were extracted and averaged across slides. Vessels chosen were ~6 μm wide (range, 5–8 μm) and 32 μm long (range of 20–55 μm). Three to five vessels that matched these criteria were chosen per ROI. The colocalization of AQP4 and GFAP was evaluated using line‐intensity scan analysis and Mander's overlap coefficients [6]. AQP4 was designated as channel 1 and GFAP as channel 2 to compute Mander's overlap coefficients. Given that this analysis specifically examined perivascular AQP4 and GFAP intensity profiles, an increase in overlap coefficients was interpreted as evidence of AQP4 polarization.

### 2.7 Electron Microscopic Morphological Analysis

Brain fragments (1 × 1 × 1 mm^3^) from the frontal cortex were fixed overnight with 2.5% glutaraldehyde at 4°C. Next, brain fragments were refixed in osmium tetroxide for 1.5 h at 4°C, dehydrated in graded ethanol, and the sections were stained with uranyl acetate and lead citrate. Digital images of the microvessels were acquired using a HITACHI HT7800 180‐kV transmission electron microscope (TEM; The Servicebio) at 1500 × and 2500 × magnification [6].

### 2.8 Statistical Analysis

Statistical analysis was performed using GraphPad Prism 10.4.0 (GraphPad Software, San Diego, CA, USA) and SPSS 27.0 (IBM Corp., Armonk, NY, USA). The Shapiro–Wilk test was used to assess the normality of continuous variables. Descriptive statistics and exploratory comparisons were conducted using Student's *t*‐test or one‐way and two‐way ANOVA. For four‐group comparisons, ordinary one‐way ANOVA was used when the assumption of equal variances was satisfied; otherwise, Welch's ANOVA was applied. Appropriate post hoc multiple‐comparison tests were conducted following significant overall effects. In analyses where the four experimental groups were analyzed as a single grouping factor, the model included only one independent factor rather than a factorial design with two or more factors. Accordingly, no interaction term was included, and interaction effects were not applicable. Statistical significance was set at *p* < 0.05. Given the small sample size, these analyses were considered exploratory and hypothesis‐generating rather than confirmatory. Accordingly, *p* values were interpreted cautiously and in conjunction with effect direction, temporal patterns, and consistency across imaging and histological readouts. MR data were presented with a 95% confidence interval (CI) and other data were represented by the mean ± standard error of the mean (SEM).

## 3 Results

### 3.1 Time‐Course Analysis of CSF‐ISF Exchange‐Related Dynamics in the Whole Brain

**FIGURE 2:**
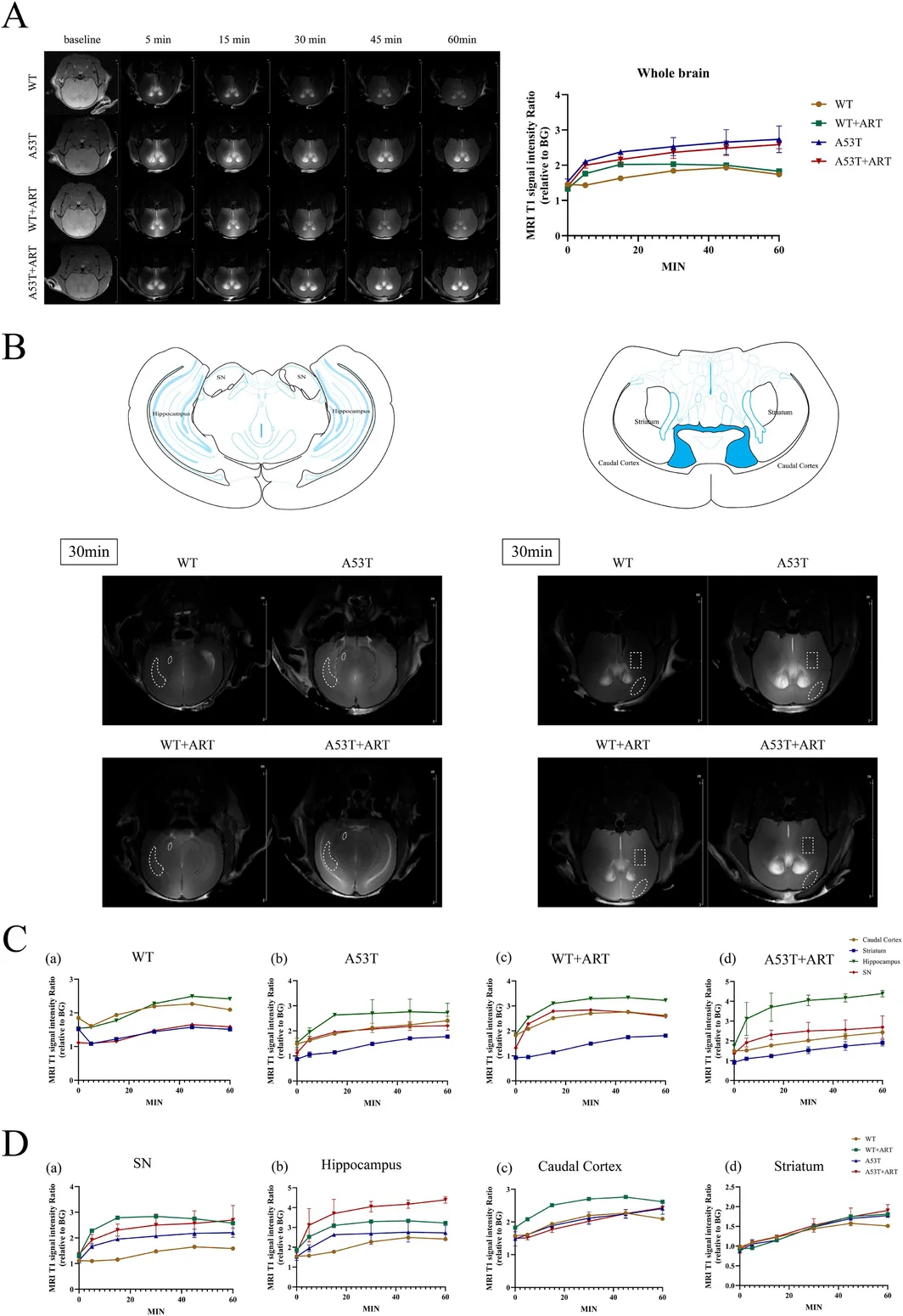
Time‐course analysis of MR contrast agent‐related to CSF tracer distribution and CSF‐ISF exchange‐related dynamics. T1‐weighted MRI signal intensity was measured in selected brain regions of the mouse brain to visualize temporal and regional changes in Gd‐DTPA‐associated tracer distribution after intraventricular administration. (A) Temporal quantification of signal intensity within the entire brain parenchyma. (B) A representative post‐contrast T1‐weighted MR image acquired 30 min after Gd‐DTPA administration illustrates regions of interest (ROIs), including the substantia nigra (SN), hippocampus, caudal cortex, and striatum. Dashed white circles delineate individual ROIs. (C) Regional signal intensity dynamics across different ROIs within each experimental group. (D) Signal intensity change trend along time within the same ROI across experimental groups. Data represent mean ± SEM. Scale bars are indicated. The scale bar in figure A, B represents 1 cm.

A trend toward reduced whole‐brain Gd‐DTPA penetration was observed in the A53T group compared to the A53T + ART group, with mean MR intensity ratios of 2.165 (A53T group, 95% CI, 1.711–2.619) and 2.321 (A53T + ART group, 95% CI, 1.850–2.792), respectively, over time. In the WT group, the mean MR intensity ratio was 1.671 (95% CI, 1.458–1.884), while the WT + ART group showed a modest increase to 1.829 (95% CI, 1.549–2.109). Figure 2A illustrates a trend toward higher MR intensity ratios in the A53T and A53T + ART groups compared to the WT and WT + ART groups, with the WT group exhibiting the lowest signal intensity across all groups over the time course.

The MR intensity of the whole brain parenchyma peaked around 45 min in both the WT and WT + ART groups, before decreasing until the 60‐min mark of the dynamic MR scan. In contrast, the MR intensity in the A53T and A53T + ART groups continued to rise through to 60‐min scan. At 5 min postcontrast injection, the WT + ART group exhibited higher intensity compared to the WT group, and this difference was maintained throughout the scan, suggesting that ART may enhance CSF‐ISF exchange‐related dynamics in this pilot dataset. Consistent with a previous study [19], the WT group showed the lowest MR intensity of the whole‐brain parenchyma across all four groups.

### 3.2 Time‐Course Analysis of CSF‐ISF Exchange‐Related Dynamics in Brain Regions

In the WT group, the MR intensity ratio in the hippocampus was 2.013 (95% CI, 1.559 to 2.466), that in the caudal cortex was 1.993 (95% CI, 1.737 to 2.249), that in the SN was 1.347 (95% CI, 1.081 to 1.612), and that in the striatum was 1.393 (95% CI, 1.188 to 1.597) over the time course (Figure 2C(a)). In the A53T group, the hippocampus showed the highest MR intensity ratio at 2.381 (95% CI, 1.836 to 2.927), followed by the caudal cortex at 1.958 (95% CI, 1.579 to 2.388), the SN (1.867; 95% CI, 1.433 to 2.301), and the striatum (1.338; 95% CI, 0.953 to 1.724) across the experimental period (Figure 2C(b)).

In the WT + ART group, the hippocampus showed the highest MR intensity ratio at 2.889 (95% CI, 2.276 to 3.502), followed by the SN at 2.425 (95% CI, 1.818 to 3.032), the caudal cortex at 2.416 (95% CI, 2.022 to 2.811), and the striatum at 1.345 (95% CI, 0.934 to 1.756) during the observation period (Figure 2C(c)). In the A53T + ART group, the hippocampus displayed the highest MR intensity ratio at 3.527 (95% CI, 2.498 to 4.557), followed by the SN at 2.225 (95% CI, 1.699 to 2.750), the caudal cortex at 1.914 (95% CI, 1.508 to 2.320), and the striatum at 1.403 (95% CI, 1.002 to 1.804) over the course of the study (Figure 2C(d)).

Figure 2D presents MR intensity ratios across four ROIs in the four animal groups, illustrating heterogeneous spatial distribution. Consistent with the MR intensity observed in the whole brain of the WT and WT + ART groups, MR intensity in the four ROIs also peaked at 45 min, except for the striatum in the WT + ART group, which showed an increasing trend until 60 min. Both the SN and hippocampus showed higher signal intensity ratios in the A53T + ART group as early as 5 min post‐injection, compared to the A53T group. This trend continued until the 60‐min mark when the MR scan concluded. Notably, the A53T + ART group exhibited a higher signal intensity ratio in the striatum at 45 min post‐dynamic MRI compared to the A53T group. While the caudal cortex showed enhanced signal change in WT mice 5 min after ART administration, no clear between‐group separation was observed in the caudal cortex and striatum between the A53T and A53T + ART groups. However, both regions exhibited an increasing trend until 60 min, rather than peaking at 45 min as seen in the WT and WT + ART groups.

### 3.3 Assessment of α‐Syn Immunofluorescence Signal Across Brain Regions

**FIGURE 3:**
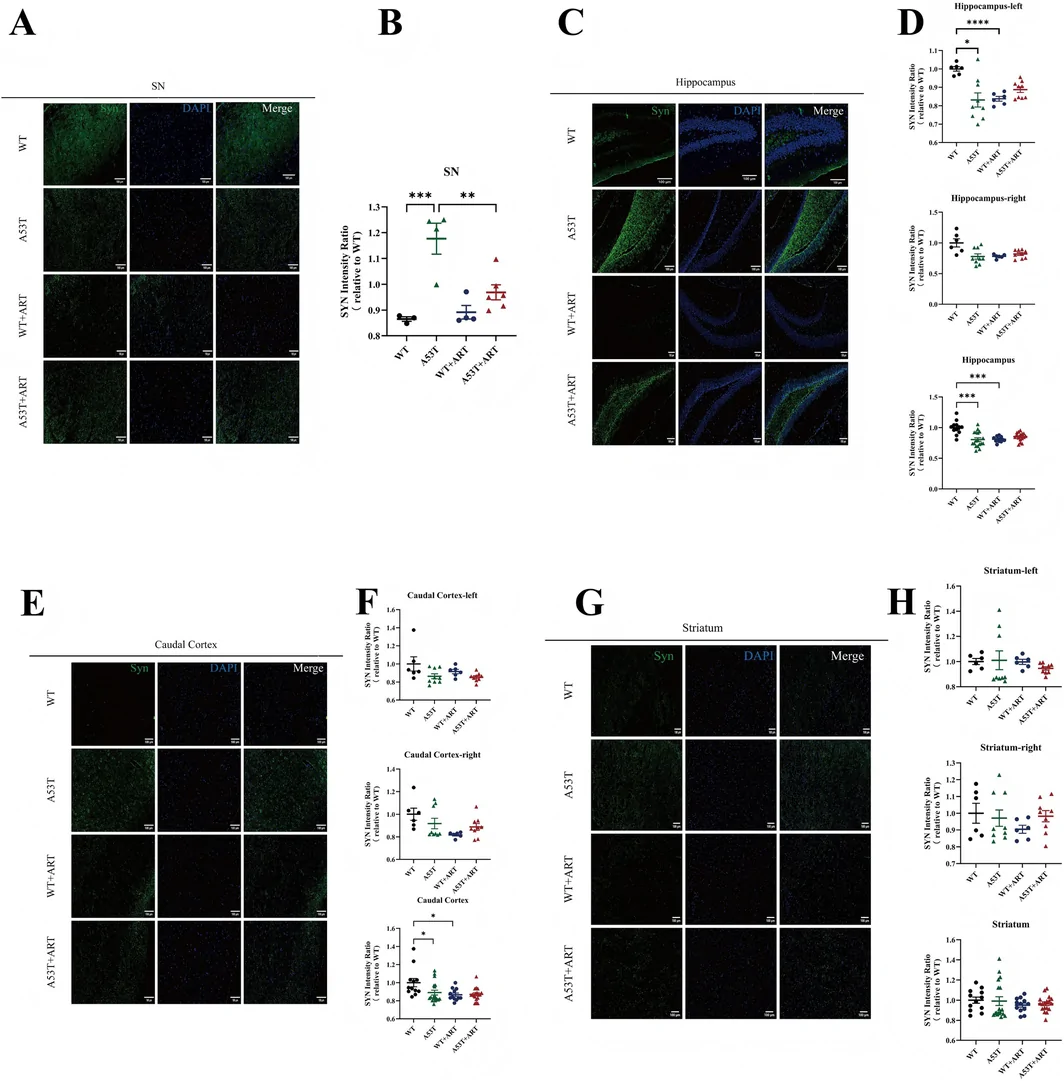
Representative immunofluorescence images of α‐syn staining (green) and the quantification of the α‐syn intensities in the SN (A, B), hippocampus (C, D), caudal cortex (E, F) and striatum (G, H) across four groups. ((B): WT and WT + ART: *N* = 4 slices from 2 animals, A53T and A53T + ART: *N* = 6 slices from 3 animals; (D, F, H): WT and WT + ART: *N* = 12 slices from 2 animals, A53T and A53T + ART: *N* = 18 slices from 3 animals). Data represent mean ± SEM.*p < 0.05; **p < 0.01; ***p < 0.001; ****p < 0.0001. The scale bar represents 100 μm.

As shown in Figure 3A,B, the A53T group exhibited significantly higher α‐syn fluorescence in the SN compared with WT (*p* = 0.0007) and the A53T+ ART group (*p* = 0.0052). A marked attenuation of α‐syn fluorescence was observed in the left hippocampus (*p* < 0.0001), bilateral hippocampi (*p* = 0.0004) (Figure 3C,D), and bilateral caudal cortex (*p* = 0.0159) (Figure 3E,F) in the WT group compared with the WT + ART group. Notably, α‐syn fluorescence in the left hippocampus was higher in the WT group than in the A53T group (*p* = 0.0102). Similarly, α‐syn fluorescence in the bilateral hippocampi (*p* = 0.0008) and caudal cortex (*p* = 0.0363) was higher in the WT group compared with the A53T (Figure 3C,D). No significant pairwise difference was observed in the striatum (Figure 3G,H). Detailed overall statistics are summarized in Table S1.

### 3.4 Assessment of AQP4 Expression Using Immunofluorescence

**FIGURE 4:**
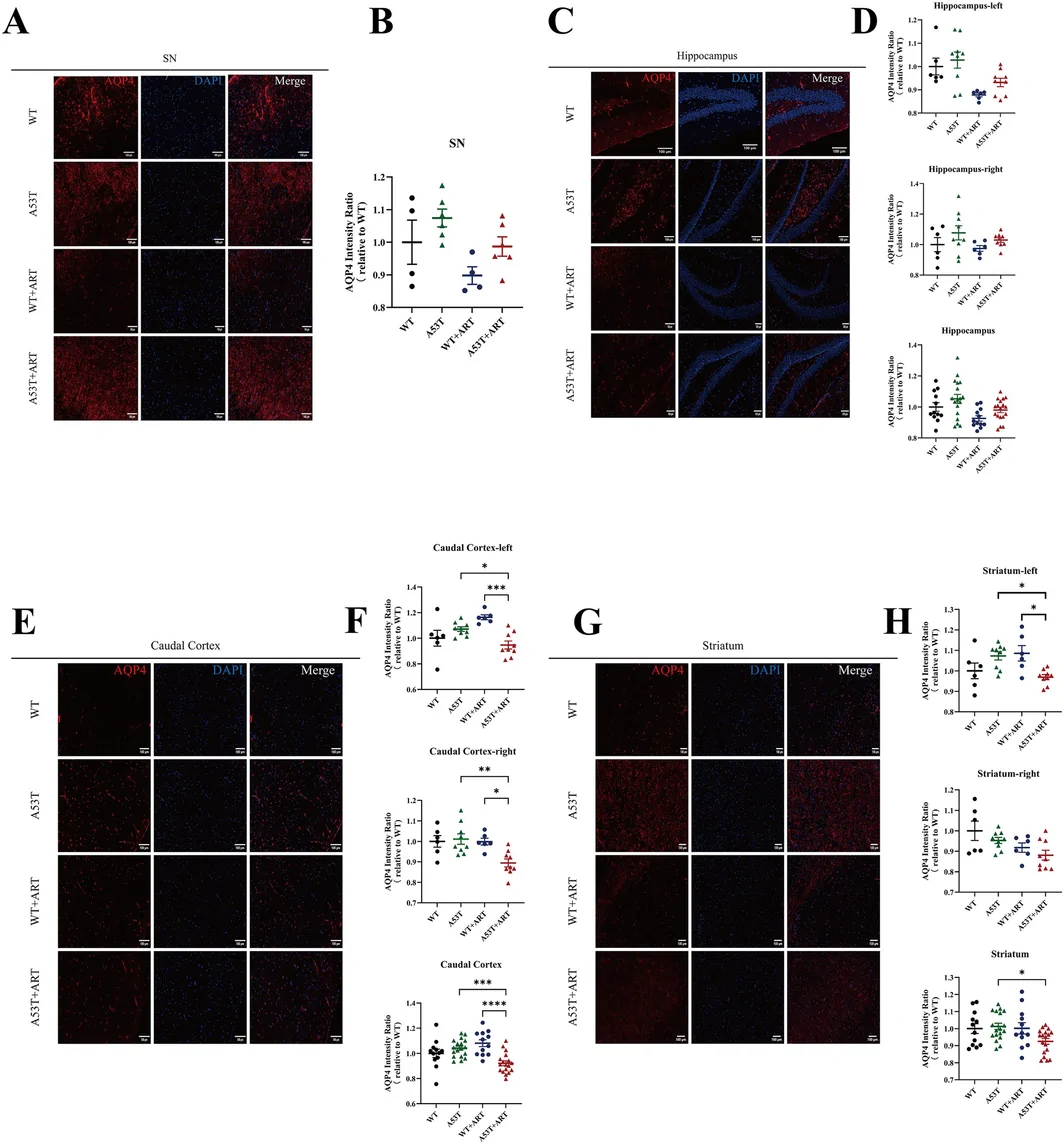
Representative immunofluorescence images of AQP4 staining (red) and the quantification of the AQP4 intensities in the SN (A, B), hippocampus (C, D), caudal cortex (E, F) and striatum (G, H) across four groups. ((B), WT and WT + ART: *N* = 4 slices from 2 animals, A53T and A53T + ART: *N* = 6 slices from 3 animals; (D, F, H), WT and WT + ART: *N* = 12 slices from 2 animals, A53T and A53T + ART: *N* = 18 slices from 3 animals). Data represent mean ± SEM.*p < 0.05; **p < 0.01; ***p < 0.001; ****p < 0.0001. The scale bar represents 100 μm.

We observed lower AQP4 in the caudal cortex (left, *p* = 0.2260; right, *p* = 0.0033; bilateral, *p* = 0.0007, Figure 4E,F) and striatum (right, NS, *p* = 0.1251; left, *p* = 0.0199; bilateral, *p* = 0.0241, Figure 4G,H) in the A53T + ART group than in the A53T group. Also, analysis revealed significantly higher AQP4 fluorescence in both the caudal cortex (left, *p* = 0.0004; right, *p* = 0.0222; bilateral, *p* < 0.0001, Figure 4F) and left striatum (*p* = 0.0194, Figure 4H) in the WT + ART group compared with the A53T + ART group. Although overall group effects were detected in the SN and in the left and bilateral hippocampi, ART‐related pairwise differences within genotype groups were not consistently observed in these regions (Figure 4A–D). Detailed overall statistics are summarized in Table S2.

### 3.5 Artesunate Restores AQP4 Polarization and Astrocyte End‐Feet Stability

**FIGURE 5:**
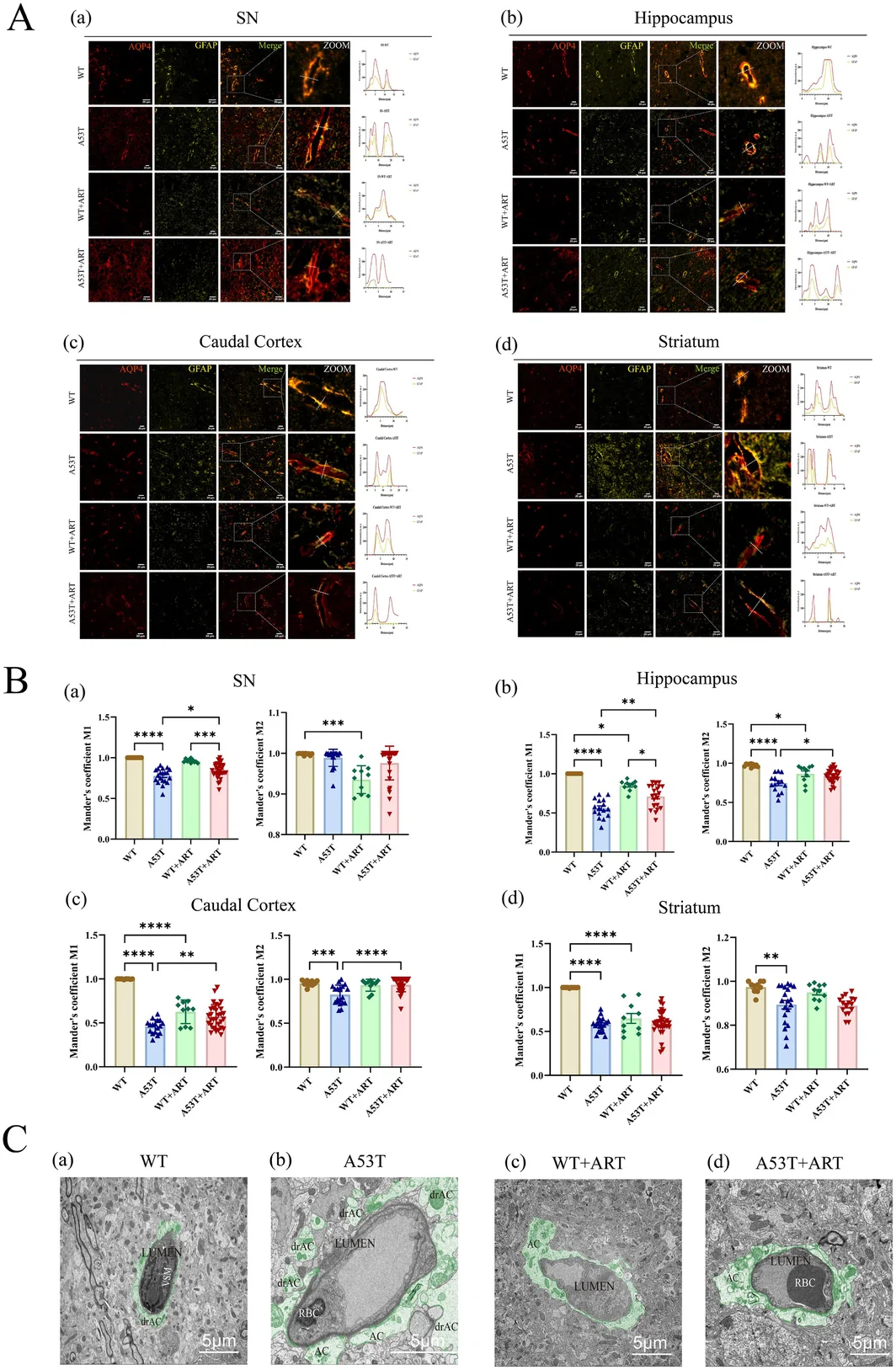
Colocalization of AQP4 with astrocyte end‐feet. (A) Representative 20 × magnification immunofluorescence images of AQP4 (red) and GFAP (yellow) in the SN (a), hippocampus (b), caudal cortex (c) and striatum (d) across four groups. White dotted squares show the colocalization of AQP4 with astrocyte end‐feet. The scale bar represents 20 μm. Line‐scan analysis was performed to analyze the colocalization. (B) Quantification of the AQP4‐ and GFAP‐ M1 and M2 Mander's overlap coefficients. (WT and WT + ART: *N* = 20 vessels from 2 animals, A53T and A53T + ART: *N* = 54–90 vessels from 3 animals) (C) Representative TEM images (a‐d) of perivascular astrocyte end‐feet (green) in the frontal cortex (*n* = 2 per group). Data are represented as mean ± SEM. *p < 0.05; **p < 0.01; ***p < 0.001; ****p < 0.0001; One‐way ANOVA with Tukey's multiple comparisons test for (B). Magnification 1500 × (a, c, d) and 2500 × (b); AC, astrocytes; drAC = detached retracted AC; VSM, vascular smooth muscle cell; RBC, red blood cell. Scale bars are indicated.

As shown in Figure 5A,B, compared with the WT group, the labeled AQP4 and GFAP were detached along the perivascular area in the SN (M1: *p* < 0.0001, Figure 5B(a)), hippocampus (M1: *p* < 0.0001; M2: *p* < 0.0001, Figure 5B(b)), caudal cortex (M1: *p* < 0.0001; M2: *p* = 0.0010, Figure 5B(c)) and striatum (M1: *p* < 0.0001; M2: *p* = 0.0060, Figure 5B(d)) in the A53T group. Furthermore, in the ART‐treated A53T group, animals exhibited enhanced detachment of labeled AQP4 and GFAP along the perivascular space in the SN (M1: *p* = 0.0004, Figure 5B(a)) and hippocampus (M1: *p* = 0.0107, Figure 5B(b)) compared with the ART‐treated WT group.

Moreover, compared with the A53T group, the A53T + ART group showed partial restoration of AQP4 and GFAP labeling along the perivascular areas in the SN (M1: *p* = 0.0281), hippocampus (M1: *p* = 0.0011; M2: *p* = 0.0117), and caudal cortex (M1: *p* = 0.0068; M2: *p* < 0.0001). However, no clear difference was detected in the striatum. Interestingly, following ART treatment, the WT group exhibited slight detachment of labeled AQP4 and GFAP along the perivascular space in the SN (M2: *p* = 0.0003), hippocampus (M1: *p* = 0.0246; M2: *p* = 0.0435), caudal cortex (M1: *p* < 0.0001), and striatum (M1: *p* < 0.0001) compared to the untreated WT group (Figure 5A,B). Detailed overall statistics are summarized in Table S3.

Ultrastructural changes in the perivascular basement membrane (BM)–astrocyte end‐feet contact in the frontal cortex were observed using TEM images and compared across the four mouse groups (Figure 5C). Compared with the WT group, the A53T group exhibited severe swelling, rounding, retraction, detachment, and partial separation of the astrocyte end‐feet from the BM. In the A53T + ART group, perivascular BM–astrocyte end‐feet contact was significantly restored, indicating that ART helps in maintaining the structural integrity of astrocytic end‐feet and their attachment to the perivascular BM.

## 4 Discussion

In this pilot study, we investigated the effects of ART on glymphatic‐related fluid transport and AQP4 in A53T transgenic PD mouse models. Our results showed that ART was associated with altered CSF tracer distribution and CSF‐ISF exchange‐related MRI dynamics, with spatial heterogeneity across brain regions. These imaging findings were directionally accompanied by decreased AQP4 expression but restored AQP4 polarization, suggesting ART's potential to modulate glymphatic‐related fluid transport. Given the small sample size, these results should be interpreted as preliminary and hypothesis‐generating rather than as definitive evidence of efficacy or mechanism.

Our findings revealed spatial heterogeneity in glymphatic‐related fluid transport in both WT and A53T mice before and after ART administration, consistent with previous studies characterizing regional brain fluid transport abnormalities in PD [26]. The spatial patterns of CSF tracer‐related MRI signal dynamics observed in our A53T mouse models align with findings from previous AD [20] and ALS models [27], suggesting a shared region‐specific feature of impaired brain fluid transport across neurodegenerative diseases. Dynamic MR illustrated that ART altered tracer‐related signal dynamics both whole‐brain‐wise and ROI‐wise in WT mice, and was associated with region‐specific changes in intraventricular CSF tracer redistribution and CSF‐ISF exchange‐related transport in the striatum, SN, and hippocampus in PD mouse models. At 5 min post‐injection, the A53T + ART group showed a higher signal intensity ratio in the SN (Figure 2D(a)) and hippocampus (Figure 2D(b)) compared with A53T. By 45 min, the signal intensity ratio in the striatum was also higher (Figure 2D(d)). The time window from 5 to 45 min post‐injection appeared to capture the most apparent between‐group signal differences in this dataset. These early imaging differences indicate that ART may influence glymphatic‐related fluid transport in PD‐vulnerable regions, potentially restoring regional function. However, this interpretation requires validation in larger cohorts.

The ex vivo findings in both WT and PD mouse models showed directional consistency with the in vivo imaging observations. Lower α‐syn immunofluorescence signal in the SN of the A53T + ART group compared with the A53T group (Figure 3A,B) corresponded with increased MR intensity in the SN in the respective groups (Figure 2D(a)). Interestingly, Tiratsuyan et al. reported region‐specific responsiveness to ART in AD rat models, with particularly heightened effects in the hippocampus, highlighting the regional specificity of ART's effects in neurodegenerative disease progression [28]. These observations were consistent with ART‐associated changes in CSF tracer redistribution and suggest a potential association between brain fluid transport‐related changes and regional patterns of α‐syn immunoreactivity, particularly in selected regions such as the SN. However, these findings should be interpreted cautiously. The higher hippocampal and cortical α‐syn fluorescence in WT compared with A53T mice does not necessarily reflect physiological α‐syn expression, as the present assay measured total α‐syn immunoreactivity rather than pathology‐specific α‐syn species. Therefore, regional differences in α‐syn immunofluorescence cannot be directly interpreted as differences in pathological α‐syn burden or clearance [29].

Interestingly, semi‐quantitative analysis of AQP4 expression revealed that ART downregulated AQP4 levels in the caudal cortex and striatum of PD mouse models, while no significant differences were observed in the SN or hippocampus across all four groups (Figure 4). In contrast, Mander's overlap coefficients showed that ART restored AQP4 polarization in the SN, hippocampus, and caudal cortex, but not in the striatum in A53T animals. ART also restored AQP4 polarization in all four regions in WT animals (Figure 5B). This regional specificity of AQP4 repolarization also directionally aligns with the spatial pattern observed in our MR imaging results (Figure 2). Since AQP4 polarization is critical for efficient perivascular fluid exchange and glymphatic‐related solute transport [5], the observed repolarization of AQP4 following ART treatment may represent one possible explanation for the ART‐associated imaging and histological changes observed in this pilot study, likely through repolarization rather than over‐expression of AQP4 in PD. This hypothesis is further supported by TEM results, which revealed improved contact between the perivascular basement membrane and astrocytic end‐feet in A53T mouse models (Figure 5C).

The regional heterogeneity in AQP4 expression and repolarization ex vivo may help explain the spatial differences in dynamic MRI tracer‐related signal changes. The lack of marked regional differences in the striatum across groups (Figure 2D(d)) is consistent with a previous report demonstrating that AQP4 expression varies across brain regions, with lower levels observed in the cerebral cortex and striatum, and the highest in the cerebellum [30]. Although a previous study on PD brains reported that AQP4 expression contributes to the reduced α‐syn deposition [9], the observed AQP4 repolarization in the SN, together with decreased α‐syn levels and increased tracer‐related MRI signal intensity ratio in our A53T mice following ART treatment, suggests that ART may be associated with restored AQP4 polarization together with altered glymphatic‐related fluid transport and reduced α‐syn immunofluorescence signal in the SN.

Notably, several ART‐associated effects were also observed in WT mice, including changes in MRI signal dynamics and AQP4/GFAP overlap. These findings raise the possibility that ART may have baseline effects on brain fluid regulation or astrocyte‐associated pathways, rather than acting only under disease conditions. This interpretation is supported by previous studies showing that glymphatic‐associated transport, perivascular fluid exchange, and astrocyte biology are modifiable under physiological as well as pathological conditions, particularly through changes in astrocyte reactivity, AQP4 localization/polarization, and neurovascular/perivascular regulation [31]. Such an interpretation is consistent with recent experimental evidence showing that glymphatic and lymphatic fluid transport are dynamically regulated even under baseline physiological conditions, including circadian‐state‐dependent changes in brain fluid exchange in healthy animals [32]. In this context, the ART‐related alterations detected in WT mice are biologically plausible and indicate that ART may exert broader modulatory effects on CSF‐ISF exchange‐related dynamics and astrocyte‐associated functions. In our study, this possibility is further supported by the regional α‐syn findings, in which the lower α‐syn accumulation in WT + ART relative to WT in the hippocampus (Figure 3C,D) and caudal cortex (Figure 3E,F) did not conform to a simple PD‐rescue pattern. Accordingly, although some changes in A53T mice remained directionally compatible with a rescue‐like effect, particularly in selected regions, the present data more cautiously support ART as a modulator of glymphatic‐associated transport and astrocyte‐related responses rather than as a purely PD‐specific rescue intervention.

ART may restores AQP4 polarization through its ability to mitigate neuroinflammation [12, 17, 33, 34, 35, 36, 37, 38] and exert antioxidative effects on astrocytes. In PD models, inflammatory activation of astrocytes not only triggers the release of inflammatory mediators but also disrupts AQP4 polarity in their end‐feet processes. By indirectly stabilizing the physiological state of astrocytes, ART promotes an environment conducive to the recovery of AQP4 polarization. Furthermore, ART has distinctive free‐radical‐scavenging capabilities and demonstrates an antioxidative effect in normal neurons and glial cells [39, 40]. By attenuating oxidative stress‐induced damage to astrocytic end‐feet, ART maintains AQP4 polar distribution, thereby sustaining the microenvironment necessary for CSF‐ISF exchange‐related transport.

Moreover, glymphatic‐related fluid transport relies on the perivascular space (PVS) as the interface between BBB capillaries and astrocytic end‐feet. ART's effects on BBB integrity likely also influence perivascular fluid transport and related CSF‐ISF exchange processes [41, 42]. ART has been shown to preserve BBB structural integrity [43], providing a stable anatomical foundation for perivascular fluid transport. ART's restoration of AQP4 polarization on astrocytic end‐feet likely contributes to the maintenance of BBB integrity. Both effects are essential for unobstructed PVS function, which is crucial for efficient brain fluid transport.

ART downregulated AQP4 expression while restoring its polarization in A53T mice, in a pattern that was aligned with previous studies that emphasize the importance of proper AQP4 polarization, rather than overexpression, for effective waste clearance [32]. Through the repolarization of aquaporins, ART enables the cellular compensatory demand for aquaporins to return to basal levels, thereby downregulating their expression via negative feedback mechanisms. Additionally, studies by Peng et al. have demonstrated that ART exerts neuroprotective effects in cortical ischemic models (e.g., reduced edema and neuroinflammation), which may partially result from indirect glymphatic‐related regulation [44]. Notably, this regulatory pattern shows striking mechanistic parallels to previously reported regulatory mechanisms of pancreatic AQP7, suggesting the existence of conserved pathological adaptation pathways within the aquaporin family [45].

Besides the neuroprotective effects, it has been demonstrated that artemisinin exhibits dose‐dependent neurotoxic effects in animal models [46, 47], which may partially explain the reduction of AQP4 polarization and expression in the SN and hippocampus in our results. This paradoxical toxicity profile highlights the need to establish precise therapeutic windows to balance anti‐inflammatory benefits with cytotoxic risks. The regional specificity of AQP4 downregulation in the SN and hippocampus suggests microenvironment‐dependent susceptibility, possibly linked to variations in astrocyte density and neuroinflammation.

Notably, increased AQP4 expression in neurodegenerative models is often associated with astrogliosis and impaired waste clearance, rather than enhanced glymphatic function [48]. Meanwhile, similar to the AQP4 polarization spatial pattern observed in our PD mouse model, impaired glymphatic function in the caudal cortex was found to correlate with tau pathology and reduced AQP4 polarization in tauopathy models [20]. Spatial consistency in AQP4 polarization dynamics was also observed in infarction‐associated areas [49]. These results demonstrate the spatial characteristics of AQP4 expression and polarization across pathological conditions before and after treatment. Thus, appropriate in vivo methods to evaluate this spatial characteristic are crucial for identifying regional glymphatic‐related dysfunction and predicting treatment outcomes. Taken together, these observations highlight the spatial characteristics of AQP4 polarization and tracer‐related MRI dynamics across pathological conditions and treatment states. They also suggest that appropriately designed in vivo imaging approaches may be useful for future studies of regional glymphatic‐related dysfunction.

Several limitations in this pilot study should be acknowledged. First, due to a limited small sample size in each group, findings in this study are preliminary and primarily descriptive. Further validation in larger cohorts is necessary. Second, because Gd‐DTPA was administered into the lateral ventricle rather than the cisterna magna, the MRI findings should not be interpreted as a definitive measure of canonical glymphatic inflow or clearance, but rather as reflecting intraventricular CSF tracer redistribution and glymphatic‐related fluid transport processes under the specific experimental conditions of this study. In addition, the MRI analysis in the present study focused on descriptive time‐course signal behavior and did not include formal kinetic modeling or a pre‐specified quantitative comparison of derived transport parameters; therefore, imaging interpretations should remain exploratory. Third, although the imaging findings were directionally supported by ex vivo observations of AQP4 polarization, astrocytic end‐feet integrity, and α‐synuclein burden, the underlying mechanisms of ART action were not directly dissected. Moreover, ART‐related changes observed in WT animals suggest that ART may exert both disease‐modifying and broader effects on brain fluid dynamics and astrocyte‐associated pathways, rather than a strictly disease‐selective rescue effect. Finally, the relatively short duration of drug administration and observation may also obscure potential long‐term effects or sustained therapeutic benefits of ART in PD mouse models. In addition, the A53T group was defined based on supplier designation without independent PCR genotyping or pre‐inclusion phenotypic stratification, and only a single 7‐day ART regimen was examined; therefore, model‐stage heterogeneity and the optimal treatment window remain to be further clarified.

## 5 Conclusion

This pilot study supports the feasibility of using dynamic contrast MR after intraventricular Gd‐DTPA administration as an exploratory in vivo readout of CSF tracer distribution and glymphatic‐related fluid transport in the A53T PD mouse model, which was further validated ex vivo with consistent findings. ART was associated with region‐specific alterations in brain fluid transport dynamics, partial restoration of AQP4 polarization, and changes in astrocyte end‐feet organization, accompanied by a reduced α‐syn immunofluorescence signal in the SN. These findings support the hypothesis that ART may modulate brain fluid transport processes relevant to the broader glymphatic framework in PD, potentially through AQP4‐related mechanisms.

## Author Contributions

(I) Conception and design: Tong Fu and Qiu Fan; (II) Administrative support: Xin‐ying Wu and Hui Xu; (III) Provision of study materials: Hui Xu, Qiu Fan, and Tong Fu; (IV) Collection and assembly of data: Wan‐ting Han, Wen‐hui Lu, Guang‐hui Xie, and Yan Cao; (V) MRI data analysis and interpretation: Wan‐ting Han, Wen‐hui Lu, and Hui Xu; (VI) Immunofluorescence and TEM data analysis and interpretation: Wan‐ting Han and Qiu Fan; (VII) Manuscript writing: Wan‐ting Han and Tong Fu; (VIII) Manuscript revision and final approval of the manuscript: All authors.

## Funding

This work was supported by Natural science foundation of Jiangsu province (BK20230155) and Nanjing Science and Technology Innovation Project Research Foundation for Returned Overseas Students to Tong Fu, Nanjing Medical University Science and Technology Development Project (No. NMUB20220061).

## Ethics Statement

Animal experiment protocols were approved by the Research Ethics Committee of the Nanjing First Hospital (Approval Number: KY20220701‐04). All procedures were carried out in strict accordance with the Guide for the Care and Use of Laboratory Animals issued by the Ministry of Science and Technology of China.

## Conflicts of Interest

The authors declare no conflicts of interest.

## Supporting information


**Table S1:** Group differences in α‐syn fluorescence across brain regions in WT, A53T, ART‐treated WT, and ART‐treated A53T mice.
**Table S2:** Group differences in AQP4 fluorescence across brain regions in WT, A53T, ART‐treated WT, and ART‐treated A53T mice.
**Table S3:** Group differences in perivascular AQP4/GFAP localization measurements across brain regions in WT, A53T, ART‐treated WT, and ART‐treated A53T mice.

## Data Availability

The data that support the findings of this study are available from the corresponding author upon reasonable request.

## References

[1] L. V. Kalia and A. E. Lang , “Parkinson's Disease,” Lancet 386, no. 9996 (2015): 896–912. 25904081 10.1016/S0140-6736(14)61393-3

[2] X. Lian , Z. Liu , Z. Gan , et al., “Targeting the Glymphatic System to Promote α‐Synuclein Clearance: A Novel Therapeutic Strategy for Parkinson's Disease,” Neural Regeneration Research 21, no. 1 (2026): 233–247. 39819820 10.4103/NRR.NRR-D-24-00764 PMC12094544

[3] L. P. Carlstrom , A. Eltanahy , A. Perry , et al., “A Clinical Primer for the Glymphatic System,” Brain 145, no. 3 (2022): 843–857. 34888633 10.1093/brain/awab428

[4] T. Bohr , P. G. Hjorth , S. C. Holst , et al., “The Glymphatic System: Current Understanding and Modeling,” iScience 25, no. 9 (2022): 104987. 36093063 10.1016/j.isci.2022.104987 PMC9460186

[5] M. M. Salman , P. Kitchen , A. Halsey , et al., “Emerging Roles for Dynamic Aquaporin‐4 Subcellular Relocalization in CNS Water Homeostasis,” Brain 145, no. 1 (2022): 64–75. 34499128 10.1093/brain/awab311 PMC9088512

[6] X. Si , S. Dai , Y. Fang , et al., “Matrix Metalloproteinase‐9 Inhibition Prevents Aquaporin‐4 Depolarization‐Mediated Glymphatic Dysfunction in Parkinson's Disease,” Journal of Advanced Research 56 (2024): 125–136. 36940850 10.1016/j.jare.2023.03.004 PMC10834796

[7] Y. Zhang , C. Zhang , X.‐Z. He , et al., “Interaction Between the Glymphatic System and Alpha‐Synuclein in Parkinson's Disease,” Molecular Neurobiology 60, no. 4 (2023): 2209–2222. 36637746 10.1007/s12035-023-03212-2

[8] S. T. Thenral and A. J. Vanisree , “Peripheral Assessment of the Genes AQP4, PBP and TH in Patients With Parkinson's Disease,” Neurochemical Research 37, no. 3 (2012): 512–515. 22083667 10.1007/s11064-011-0637-5

[9] A. Hoshi , A. Tsunoda , M. Tada , M. Nishizawa , Y. Ugawa , and A. Kakita , “Expression of Aquaporin 1 and Aquaporin 4 in the Temporal Neocortex of Patients With Parkinson's Disease,” Brain Pathology 27, no. 2 (2017): 160–168. 26919570 10.1111/bpa.12369 PMC8028881

[10] P. Newton , Y. Suputtamongkol , P. Teja‐Isavadharm , et al., “Antimalarial Bioavailability and Disposition of Artesunate in Acute Falciparum Malaria,” Antimicrobial Agents and Chemotherapy 44, no. 4 (2000): 972–977. 10722499 10.1128/aac.44.4.972-977.2000 PMC89800

[11] E. Kiss , S. Kins , K. Gorgas , K. H. Venczel Szakács , J. Kirsch , and J. Kuhse , “Another Use for a Proven Drug: Experimental Evidence for the Potential of Artemisinin and Its Derivatives to Treat Alzheimer's Disease,” International Journal of Molecular Sciences 25, no. 8 (2024): 4165. 38673751 10.3390/ijms25084165 PMC11049906

[12] W. E. Ho , H. Y. Peh , T. K. Chan , and W. S. F. Wong , “Artemisinins: Pharmacological Actions Beyond Anti‐Malarial,” Pharmacology & Therapeutics 142, no. 1 (2014): 126–139. 24316259 10.1016/j.pharmthera.2013.12.001

[13] S. Zuo , Q. Li , X. Liu , et al., “The Potential Therapeutic Effects of Artesunate on Stroke and Other Central Nervous System Diseases,” BioMed Research International 2016 (2016): 1489050. 28116289 10.1155/2016/1489050 PMC5223005

[14] Y. Liu , W. Dang , S. Zhang , L. Wang , and X. Zhang , “Artesunate Attenuates Inflammatory Injury and Inhibits the NF‐κB Pathway in a Mouse Model of Cerebral Ischemia,” Journal of International Medical Research 49, no. 11 (2021): 3000605211053549. 34743632 10.1177/03000605211053549 PMC8579345

[15] G. Xie , Y. Liang , W. Gao , et al., “Artesunate Alleviates Intracerebral Haemorrhage Secondary Injury by Inducing Ferroptosis in M1‐Polarized Microglia and Suppressing Inflammation Through AMPK/mTORC1/GPX4 Pathway,” Basic & Clinical Pharmacology & Toxicology 132, no. 5 (2023): 369–383. 36815716 10.1111/bcpt.13848

[16] H. S. Lim and G. Park , “Artemisinin Protects Dopaminergic Neurons Against 1‐Methyl‐4‐Phenyl‐1,2,3,6‐Tetrahydropyridine‐Induced Neurotoxicity in a Mouse Model of Parkinson's Disease,” Biomedicine & Pharmacotherapy 170 (2024): 115972. 38056239 10.1016/j.biopha.2023.115972

[17] J. Lv , J. Zhu , P. Wang , et al., “Artemisinin Exerts a Protective Effect in the MPTP Mouse Model of Parkinson's Disease by Inhibiting Microglial Activation via the TLR4/Myd88/NF‐KB Pathway,” CNS Neuroscience & Therapeutics 29, no. 4 (2023): 1012–1023. 36691817 10.1111/cns.14063 PMC10018080

[18] S. L. DeVos and T. M. Miller , “Direct Intraventricular Delivery of Drugs to the Rodent Central Nervous System,” Journal of Visualized Experiments 75 (2013): e50326. 10.3791/50326 PMC3679837 23712122

[19] N. Percie du Sert , V. Hurst , A. Ahluwalia , et al., “The ARRIVE Guidelines 2.0: Updated Guidelines for Reporting Animal Research,” PLoS Biology 18, no. 7 (2020): e3000410. 32663219 10.1371/journal.pbio.3000410 PMC7360023

[20] I. F. Harrison , O. Ismail , A. Machhada , et al., “Impaired Glymphatic Function and Clearance of Tau in an Alzheimer's Disease Model,” Brain 143, no. 8 (2020): 2576–2593. 32705145 10.1093/brain/awaa179 PMC7447521

[21] K. B. J. Franklin and G. Paxinos , The Mouse Brain in Stereotaxic Coordinates (Elsevier Science, 2019).

[22] G. Marino , F. Campanelli , G. Natale , et al., “Intensive Exercise Ameliorates Motor and Cognitive Symptoms in Experimental Parkinson's Disease Restoring Striatal Synaptic Plasticity,” Science Advances 9, no. 28 (2023): eadh1403. 37450585 10.1126/sciadv.adh1403 PMC10348672

[23] S. Feng , C. Wu , P. Zou , et al., “High‐Intensity Interval Training Ameliorates Alzheimer's Disease‐Like Pathology by Regulating Astrocyte Phenotype‐Associated AQP4 Polarization,” Theranostics 13, no. 10 (2023): 3434–3450. 37351177 10.7150/thno.81951 PMC10283053

[24] O. Genel , C. M. Pariante , and A. Borsini , “The Role of AQP4 in the Pathogenesis of Depression, and Possible Related Mechanisms,” Brain, Behavior, and Immunity 98 (2021): 366–377. 34474133 10.1016/j.bbi.2021.08.232

[25] K. Leng , E. Li , R. Eser , et al., “Molecular Characterization of Selectively Vulnerable Neurons in Alzheimer's Disease,” Nature Neuroscience 24, no. 2 (2021): 276–287. 33432193 10.1038/s41593-020-00764-7 PMC7854528

[26] L. Liu , Q. Weng , Q. Cai , et al., “Choroid Plexus Enlargement Contributes to Motor Severity via Regional Glymphatic Dysfunction in Parkinson's Disease,” NPJ Parkinsons Dis 11, no. 1 (2025): 134. 40404762 10.1038/s41531-025-00971-8 PMC12098873

[27] A. Zamani , A. K. Walker , and D. K. Wright , “Glymphatic Dysfunction and Neurodegeneration in ALS: Longitudinal Insights From rNLS8 TDP‐43 Mice,” Neurobiology of Disease 206 (2025): 106832. 39914774 10.1016/j.nbd.2025.106832

[28] S. Tiratsuyan , Y. Hambardzumyan , M. Poghosyan , M. Danielyan , and A. Hovhannisyan , “Neuroprotective Effect of Artemisinin in an Animal Model of Alzheimer's Disease,” Current Medicinal Chemistry 32 (2024): 6290–6305. 10.2174/0109298673312842241003111836 39428937

[29] I. Frigerio , M. M. A. Bouwman , R. T. G. M. M. Noordermeer , et al., “Regional Differences in Synaptic Degeneration Are Linked to Alpha‐Synuclein Burden and Axonal Damage in Parkinson's Disease and Dementia With Lewy Bodies,” Acta Neuropathologica Communications 12, no. 1 (2024): 4. 38173031 10.1186/s40478-023-01711-w PMC10765668

[30] J. A. Hubbard , M. S. Hsu , M. M. Seldin , and D. K. Binder , “Expression of the Astrocyte Water Channel Aquaporin‐4 in the Mouse Brain,” ASN Neuro 7, no. 5 (2015): 1759091415605486. 26489685 10.1177/1759091415605486 PMC4623559

[31] B. A. Plog and M. Nedergaard , “The Glymphatic System in Central Nervous System Health and Disease: Past, Present, and Future,” Annual Review of Pathology 13 (2018): 379–394. 10.1146/annurev-pathol-051217-111018 PMC5803388 29195051

[32] L. M. Hablitz , V. Plá , M. Giannetto , et al., “Circadian Control of Brain Glymphatic and Lymphatic Fluid Flow,” Nature Communications 11, no. 1 (2020): 4411. 10.1038/s41467-020-18115-2 PMC7468152 32879313

[33] M. Huai , J. Zeng , and W. Ge , “Artemisinin Ameliorates Intestinal Inflammation by Skewing Macrophages to the M2 Phenotype and Inhibiting Epithelial‐Mesenchymal Transition,” International Immunopharmacology 91 (2021): 107284. 33359851 10.1016/j.intimp.2020.107284

[34] J. Q. Shi , C. C. Zhang , X. L. Sun , et al., “Antimalarial Drug Artemisinin Extenuates Amyloidogenesis and Neuroinflammation in APPswe/PS1dE9 Transgenic Mice via Inhibition of Nuclear Factor‐kappaB and NLRP3 Inflammasome Activation,” CNS Neuroscience & Therapeutics 19, no. 4 (2013): 262–268. 23406388 10.1111/cns.12066 PMC6493386

[35] Z. Shi , Y. Chen , C. Lu , et al., “Resolving Neuroinflammation, the Therapeutic Potential of the Anti‐Malaria Drug Family of Artemisinin,” Pharmacological Research 136 (2018): 172–180. 30196102 10.1016/j.phrs.2018.09.002

[36] D. Wang , J. Shi , S. Lv , et al., “Artesunate Attenuates Lipopolysaccharide‐Stimulated Proinflammatory Responses by Suppressing TLR4, MyD88 Expression, and NF‐κB Activation in Microglial Cells,” Inflammation 38, no. 5 (2015): 1925–1932. 26002587 10.1007/s10753-015-0172-7

[37] L. Xia , Y. Qiu , J. Li , M. Xu , and Z. Dong , “The Potential Role of Artemisinins Against Neurodegenerative Diseases,” American Journal of Chinese Medicine 52, no. 6 (2024): 1641–1660. 39343990 10.1142/S0192415X24500642

[38] X. Zhao , X. Huang , C. Yang , et al., “Artemisinin Attenuates Amyloid‐Induced Brain Inflammation and Memory Impairments by Modulating TLR4/NF‐kappaB Signaling,” International Journal of Molecular Sciences 23, no. 11 (2022): 6354. 35683033 10.3390/ijms23116354 PMC9181281

[39] X. Zhao , J. Fang , S. Li , et al., “Artemisinin Attenuated Hydrogen Peroxide (H(2)O(2))‐Induced Oxidative Injury in SH‐SY5Y and Hippocampal Neurons via the Activation of AMPK Pathway,” International Journal of Molecular Sciences 20, no. 11 (2019): 2680. 31151322 10.3390/ijms20112680 PMC6600327

[40] W. G. Yang , A. Sun , R. Zhu , N. Liu , W. J. He , and L. L. Liu , “Exploration of Artemisinin Against IgA Nephropathy via AKT/Nrf2 Pathway by Bioinformatics and Experimental Validation,” Drug Design, Development and Therapy 17 (2023): 1679–1697. 37309415 10.2147/DDDT.S403422 PMC10257916

[41] Q. Yang , X. Wei , B. Deng , et al., “Cerebral Small Vessel Disease Alters Neurovascular Unit Regulation of Microcirculation Integrity Involved in Vascular Cognitive Impairment,” Neurobiology of Disease 170 (2022): 105750. 35580816 10.1016/j.nbd.2022.105750

[42] Y. Gao , K. Liu , and J. Zhu , “Glymphatic System: An Emerging Therapeutic Approach for Neurological Disorders,” Frontiers in Molecular Neuroscience 16 (2023): 1138769. 37485040 10.3389/fnmol.2023.1138769 PMC10359151

[43] S. Zuo , H. Ge , Q. Li , et al., “Artesunate Protected Blood‐Brain Barrier via Sphingosine 1 Phosphate Receptor 1/Phosphatidylinositol 3 Kinase Pathway After Subarachnoid Hemorrhage in Rats,” Molecular Neurobiology 54, no. 2 (2017): 1213–1228. 26820677 10.1007/s12035-016-9732-6

[44] T. Peng , S. Li , L. Liu , et al., “Artemisinin Attenuated Ischemic Stroke Induced Cell Apoptosis Through Activation of ERK1/2/CREB/BCL‐2 Signaling Pathway In Vitro and In Vivo,” International Journal of Biological Sciences 18, no. 11 (2022): 4578–4594. 35864966 10.7150/ijbs.69892 PMC9295073

[45] B. A. Khattab , M. O. Hammad , Z. H. Eldken , D. Hellal , S. Z. Mohamed , and N. H. Sakr , “Impact of Intermittent Fasting Versus Vitamin D on High Fat Fructose‐Induced Pancreatic Steatosis: Possible Role of Aquaporins,” Molecular Medicine 31, no. 1 (2025): 207. 40419958 10.1186/s10020-025-01239-w PMC12107854

[46] A. Nontprasert , S. Pukrittayakamee , M. Nosten‐Bertrand , S. Vanijanonta , and N. J. White , “Studies of the Neurotoxicity of Oral Artemisinin Derivatives in Mice,” American Journal of Tropical Medicine and Hygiene 62, no. 3 (2000): 409–412. 11037787 10.4269/ajtmh.2000.62.409

[47] D. L. Wesche , M. A. DeCoster , F. C. Tortella , and T. G. Brewer , “Neurotoxicity of Artemisinin Analogs In Vitro,” Antimicrobial Agents and Chemotherapy 38, no. 8 (1994): 1813–1819. 7986012 10.1128/aac.38.8.1813 PMC284641

[48] D. M. Zeppenfeld , M. Simon , J. D. Haswell , et al., “Association of Perivascular Localization of Aquaporin‐4 With Cognition and Alzheimer Disease in Aging Brains,” JAMA Neurology 74, no. 1 (2017): 91–99. 27893874 10.1001/jamaneurol.2016.4370

[49] J. Zhu , J. Mo , K. Liu , et al., “Glymphatic System Impairment Contributes to the Formation of Brain Edema After Ischemic Stroke,” Stroke 55, no. 5 (2024): 1393–1404. 38533660 10.1161/STROKEAHA.123.045941

