# Supplementary material for: A Pilot Study Evaluating the Effect of Artesunate on Glymphatic‐Related Fluid Transport in Parkinson's Disease Mouse Models In Vivo and Ex Vivo
Source: CNS Neurosci Ther. 2026 Sep 16;32(9):e71145. doi: 10.1002/cns.71145 (PMC13580350; doi:10.1002/cns.71145)
Supplement: Supplementary file 1 [file CNS-32-e71145-s001.docx]

**Supplementary Table 1. Group differences in α-syn fluorescence across brain regions in WT, A53T, ART-treated WT, and ART-treated A53T mice**

| **Brain region** | **Side** | **df_1_** | **df_2_** | **F** | **Welch’s W** | **P value** |
| --- | --- | --- | --- | --- | --- | --- |
| SN | bilateral | 3 | 16 | 12.66 |  | 0.0004^***^ |
| Hippocampus | left | 3 | 14.21 |  | 27.75 | < 0.0001^****^ |
|  | right | 3 | 12.69 |  | 3.637 | 0.0429^*^ |
|  | bilateral | 3 | 10.50 |  | 28.58 | < 0.0001^****^ |
| Caudal Cortex | left | 3 | 12.04 |  | 2.267 | 0.1328 |
|  | right | 3 | 26 | 2.934 |  | 0.0521 |
|  | bilateral | 3 | 56 | 4.480 |  | 0.0069^**^ |
| Striatum | left | 3 | 12.65 |  | 2.108 | 0.1499 |
|  | right | 3 | 26 | 0.7452 |  | 0.5349 |
|  | bilateral | 3 | 56 | 0.4512 |  | 0.7175 |

α-syn, α-synuclein; SN, substantia nigra; df1, numerator degrees of freedom; df2, denominator degrees of freedom; F, one-way ANOVA assuming of homogeneity of variance; Welch's W, Welch's ANOVA used when the assumption of homogeneity of variance was not met; ^*^p < 0.05; ^**^p < 0.01; ^***^p < 0.001; ^****^p < 0. 0001.

**Supplementary Table 2. Group differences in AQP4 fluorescence across brain regions in WT, A53T, ART-treated WT, and ART-treated A53T mice**

| **Brain region** | **Side** | **df_1_** | **df_2_** | **F** | **Welch’s W** | **P value** |
| --- | --- | --- | --- | --- | --- | --- |
| SN | bilateral | 3 | 7.833 |  | 6.057 | 0.0193^*^ |
| Hippocampus | left | 3 | 12.46 |  | 9.542 | 0.0015^**^ |
|  | right | 3 | 26 | 1.551 |  | 0.2250^*^ |
|  | bilateral | 3 | 28.86 |  | 5.100 | 0.0059^**^ |
| Caudal Cortex | left | 3 | 12.71 |  | 12.01 | 0.0005^***^ |
|  | right | 3 | 26 | 6.320 |  | 0.0023^**^ |
|  | bilateral | 3 | 56 | 9.590 |  | < 0.0001^****^ |
| Striatum | left | 3 | 26 | 5.050 |  | 0.0069^**^ |
|  | right | 3 | 26 | 3.492 |  | 0.0298^*^ |
|  | bilateral | 3 | 56 | 3.483 |  | 0.0216^*^ |

AQP4, aquaporin-4; SN, substantia nigra; df1, numerator degrees of freedom; df2, denominator degrees of freedom; F, one-way ANOVA assuming of homogeneity of variance; Welch's W, Welch's ANOVA used when the assumption of homogeneity of variance was not met; ^*^p < 0.05; ^**^p < 0.01; ^***^p < 0.001; ^****^p < 0. 0001.

| **Brain region** | **Manders' coefficient** | **df_1_** | **df_2_** | **F** | **P value** |
| --- | --- | --- | --- | --- | --- |
| SN | M1 | 3 | 66 | 24.78 | < 0.0001^****^ |
|  | M2 | 3 | 66 | 7.882 | 0.0001^***^ |
| Hippocampus | M1 | 3 | 55 | 33.91 | < 0.0001^****^ |
|  | M2 | 3 | 55 | 12.80 | < 0.0001^****^ |
| Caudal Cortex | M1 | 3 | 63 | 51.34 | < 0.0001^****^ |
|  | M2 | 3 | 63 | 9.135 | < 0.0001^****^ |
| Striatum | M1 | 3 | 66 | 32.37 | < 0.0001^****^ |
|  | M2 | 3 | 66 | 6.144 | 0.0012^**^ |

**Supplementary Table 3. Group differences in perivascular AQP4/GFAP localization measurements across brain regions in WT, A53T, ART-treated WT,**

**and ART-treated A53T mice**

AQP4, aquaporin-4; SN, substantia nigra; M1, Manders' colocalization coefficient representing the proportion of AQP4 signal overlapping with GFAP; M2, Manders' colocalization coefficient representing the proportion of GFAP signal overlapping with AQP4; df1, numerator degrees of freedom; df2, denominator degrees of freedom; F, one-way ANOVA assuming of homogeneity of variance; ^*^p < 0.05; ^**^p < 0.01; ^***^p < 0.001; ^****^p < 0. 0001.
